# Expression Levels and Clinical Significance of miR-21-5p, miR-let-7a, and miR-34c-5p in Laryngeal Squamous Cell Carcinoma

**DOI:** 10.1155/2017/3921258

**Published:** 2017-08-31

**Authors:** Massimo Re, Giuseppe Magliulo, Federico M. Gioacchini, Arisa Bajraktari, Andrea Bertini, Artan Çeka, Corrado Rubini, Luigi Ferrante, Antonio D. Procopio, Fabiola Olivieri

**Affiliations:** ^1^Department of Otorhinolaryngology, Umberto I University General Hospital, Università Politecnica delle Marche, Via Conca 71, 60126 Ancona, Italy; ^2^Department of Otorhinolaryngology, Audiology and Phoniatrics “G. Ferreri”, University La Sapienza, Piazzale Aldo Moro 5, 00185 Rome, Italy; ^3^Department of Molecular and Clinical Sciences, Università Politecnica delle Marche, Via Tronto 10/a, 60126 Ancona, Italy; ^4^Pathologic Anatomy and Histopathology Division, Department of Biomedical Sciences and Public Health, Università Politecnica delle Marche, Via Conca 71, 60126 Ancona, Italy; ^5^Department of Biomedical Sciences and Public Health, Section of Medical Statistics, Faculty of Medicine, Università Politecnica delle Marche, Via Tronto 10/a, 60126 Ancona, Italy; ^6^Center of Clinical Pathology and Innovative Therapy, INRCA-IRCCS Italian National Institute, Via della Montagnola 81, 60127 Ancona, Italy

## Abstract

**Objective:**

Altered microRNAs (miRNAs) expression has been found in many cancer types, including laryngeal squamous cell carcinoma (LSCC). The aim of this study was to determine the role and clinical value of three LSCC-related miRs, such as miR-21-5p, miR-let-7a, and miR-34c-5p in a homogeneous cohort of patients with primary LSCC treated by primary surgery.

**Methods:**

Expression levels of miR-21-5p, miR-let-7a, and miR-34c-5p were detected in 43 pairs of LSCC and adjacent normal tissues by reverse-transcription quantitative PCR. Overall survival and disease-free survival were evaluated using the Kaplan–Meier method, and multivariate analysis was performed using the Cox proportional hazard analysis.

**Results:**

miR-21-5p is significantly upregulated, while miR-let-7a is significantly downregulated in LSCC tumor tissues compared with the corresponding adjacent normal tissues. The downregulation of miR-34c-5p expression significantly correlated with a shorter disease-free survival and, in the multivariate analysis, low miR-34c-5p expression was associated with an increased risk of recurrence.

**Conclusions:**

miR-21-5p, miR-let-7a, and miR-34c-5p seem to play a critical role in LSCC carcinogenesis and might have a diagnostic and prognostic clinical value. The miR-let-7a levels could have a predictive role for lymph node metastases and miR-34c-5p might be a promising biomarker of patient outcome.

## 1. Introduction

Laryngeal squamous cell carcinoma (LSCC) is estimated to account for about 1.5% of all cancers and is the second most common head and neck malignancy for adults [1]. LSCC treatment planning depends on the TNM staging system; it is a globally accepted description for cancer anatomical extent but does not take into account individual tumor biology cases.

LSCCs show heterogeneous biological behaviours in recurrence and/or metastatic disease development. Therefore, patients with identical clinicopathological features may experience different clinical courses or responses to identical therapies. There is therefore a need of early diagnosis with a clinical staging system which includes relevant prognostic factors for the development of tailored, individual therapies. A number of studies have proven that epigenetic alterations of the regulatory epigenetic mechanisms play a pivotal role in head and neck squamous cell carcinoma (HNSCC) carcinogenesis [2–4]. MicroRNAs (miRNAs), a class of small non-protein-coding RNA molecules that control target gene expression, are now considered crucial components of the epigenome, responsible for events ranging from organogenesis to immunity, and critical to the development of many diseases, including cancer [5–7]. Interestingly, the overall miRNA expression profile in normal tissue is different from tumor tissue [8]. MiRNA's regulation is compromised in cancer, and a number of studies have suggested that this dysregulation may be associated with tumor characteristics and prognosis in a variety of types of tumor [9–12]. Aberrations in miRNA expression in primary HNSCC tumors have recently been defined [13–16], and we previously demonstrated the relationship of miR-34c-5p to LSCC recurrence [17]. Therefore, the expression of 3 distinct miRs, including miR-21-5p, miR-let-7a, and miR-34c-5p, in a homogeneous cohort of patients with primary LSCC treated with primary surgery was undertaken. The novel scientific content of the present manuscript is their simultaneous analysis for the evaluation of their potential differential prognostic and diagnostic significance in LSCC carcinogenesis. miR-34c-5p expression levels are compared with miR-21-5p and miR-let-7a, previously not included in LSCC analyses.

## 2. Materials and Methods

### 2.1. Selection of Patients and Specimen

The enrolment inclusion and exclusion criteria for this retrospective cohort study of patients with primary LSCC diagnoses, consecutively treated with primary surgery at the Department of Otorhinolaryngology, Umberto I University General Hospital,* Università Politecnica delle Marche*, Ancona, Italy, between 1999 and 2004, have been previously described [17].

### 2.2. Patient Cohort

The relevant clinicopathologic features of the 43 patients who met the inclusion criteria are outlined in Table 1. Locoregional disease without distant metastases was registered for all 43 patients. Curative surgical resection was prescribed, followed by postoperative radiotherapy and/or chemotherapy in some cases of T3-T4 tumors and in all cases of N2 disease or extracapsular lymph node spread (ECS).

The cohort included 42 (97.67%) males with a mean age of 66.5 years (range 54–84). Tumors were classified as transglottic (*n* = 33, 76.7%), supraglottic (*n* = 8, 18.6%), and subglottic (*n* = 2, 4.7%). According to the American Joint Committee on Cancer (AJCC) TNM Staging System for the Larynx (7th ed., 2010), 31 patients were classified as stage III (72.1%) and 12 as stage IV (27.9%).

Most patients were treated with a total laryngectomy (*n* = 33, 76.7%) and some with a partial laryngectomy (*n* = 10, 23.3%). Of the partial laryngectomies performed, 7 were supraglottic and 3 were crico-hyoido-pexie (CHP). Most patients received a monolateral or bilateral neck dissection (*n* = 33, 77%). Postoperative radiotherapy was prescribed for most patients (*n* = 22, 51%) and radiochemotherapy was prescribed for 10 patients (23%). Histopathological results revealed 5 well-differentiated (Grade 1), 29 moderately differentiated (Grade 2), and 9 poorly differentiated (Grade 3) tumors. None of the tumors were registered in or close to (<1 mm) the surgical margins or the extracapsular lymph node spread (ECS).

### 2.3. Follow-Up

At a mean follow-up of 55.7 months (range 6–168 months; SD = 45.7), 23 patients (53.5%) recorded tumor relapse and subsequently died from LSCC. Four patients died from unrelated causes. Of the 20 (46.5%) surviving patients, there was no evidence of active disease at the final follow-up visit.

### 2.4. RNA Extraction and Analysis of miRNA Expression

MicroRNA- (miR-) 34c-5p, miR-21-5p, and miR-let7a were selected according to data in literature supporting their dysregulation in LSCC [18–20].

RNA extraction from FFPE samples and quantification has been previously described [17]. RNU48 was used for qRT-PCR data normalization. The relative miR expression was calculated as follows: 2^−DCt^, DCt = Ct miR-X − Ct RNU, and it was reported as arbitrary units (a.u.).

Ct values from qRT-PCR assays > 35 were considered as not expressed. The intra- and interassay variability of miR measurements were <5% and <10%, respectively.

### 2.5. Statistical Analysis

miR-34c-5p, miR-21-5p, and miR-let7a expression levels were measured in 43 samples of both tumor and healthy tissue, and the presence of lymph node metastasis *N* (absence *N* = 0 and presence *N* = 1 or *N* = 2), tumor grade of differentiation G (G1 versus G2 or G3), and presence or absence of recurrence were registered.

The distributions of each miRNA between the different groups were compared using the Mann–Whitney test. Both the diagnostic and prognostic accuracy of each miR and the ratio between the three miRs were analysed with the Receiver Operating Characteristic (ROC) curve. The area under the ROC curve (AUC) was used as a diagnostic index. Overall survival (OS) is defined as time from surgery until death from any cause or the latest clinical follow-up, and disease-free survival is defined as time from treatment completion until disease relapse. The Kaplan–Meier (KM) method estimated the cumulative incidence function (CIF) of disease-free survival and OS. The CIFs for different groups were compared by log-rank statistic. KM curves were created according to miR levels and based on the ROC best cut-off value calculations (i.e., the maximum sum of miR level specificity and sensitivity), lymph node metastasis pN (absence pN = 0 and presence pN > 0), and tumor grade of differentiation G (G1 or G2 versus G3). For estimates of LSCC recurrence incidence, patients without relapse at their latest clinical follow-up were censored. A Cox proportional hazard model was used for the multivariate analysis assessing the effect of prognostic factors on disease-free survival and OS. The Akaike information criterion (AIC), with an appropriate partial likelihood, was constructed for model comparisons and for the best model selection (fit for several models to determine the lowest AIC). The statistical software R was used for all statistical analyses [21]. Significance was considered <0.05 and all reported *p* values were two-tailed.

## 3. Results

**Figure 1:**
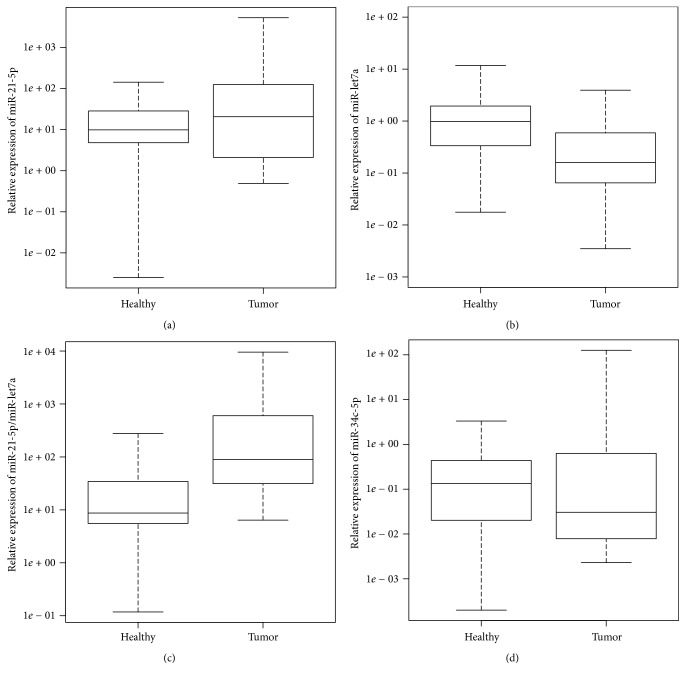
miR-21-5p (a), miR-let7a (b), the miR-21-5p/miR-let7a ratio (c), and miR-34c-5p (d) relative expression in healthy, laryngeal samples and LSCC samples.

The expression of miR-21-5p in LSCC tissue was significantly higher than in normal adjacent tissue (median and interquartile range, Me = 20.64 and IQR = 111.96 versus Me = 9.85 and IQR = 23.63, *p* = 0.037), whereas miR-let7a expression in LSCC was significantly lower than in adjacent normal tissue (Me = 0.160 and IQR = 0.528 versus Me = 0.986 and IQR = 1.609, *p* < 0.001) (Figure 1).

To improve the predictive potential for individual microRNA alterations, we used expression ratios as described by Gordon et al. [22]. The ratio of miR-21-5p/miR-let7a (Figure 1) showed significant discriminatory potential in distinguishing tumor tissue from normal adjacent tissue (Me = 89.91 and IQR = 570.56 versus Me = 8.72 and IQR = 28.84, *p* < 0.01). On the contrary, mir-34c-5p expression was not significantly different between LSCC and normal tissue (Me = 0.031 and IQR = 0.565 versus Me = 0.134 and IQR = 0.418, *p* = 0.99).

**Figure 2:**
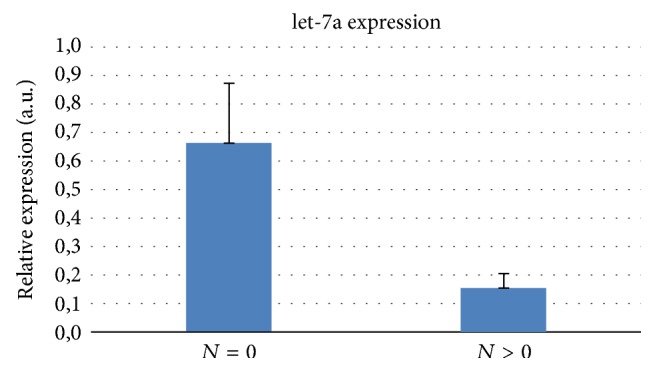
miR-let-7a and nodal involvement relative expression (*N*).

The downregulation of miR-let-7a was statistically significant in tumor sample subgroups divided according to the pN values reaching the minimum levels of expression in samples with pN > 0 (mean ± error standard *N* = 0 versus *N* > 0, let-7a: 0.666 ± 0.210 versus 0.155 ± 0.05, *p* = 0.027). These data suggest that miR-let-7a levels could have a predictive role in lymph node metastases (Figure 2).

**Figure 3:**
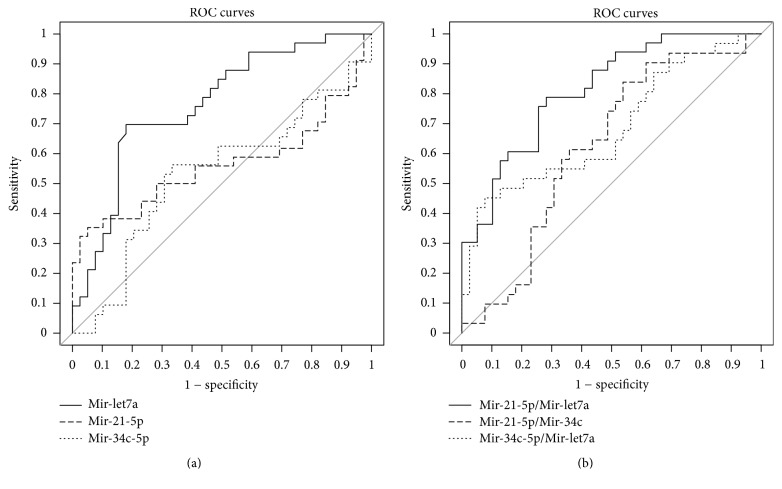
Sensitivity and specificity of miR-21-5p, miR-34c-5p, and miR-let7a (a) and miR-21-5p/miR-let7a, miR-21-5p/miR-34c-5p, and miR-34c-5p/miR-let7a (b) in discriminating healthy from LSCC samples, according to the Receiver Operating Characteristic (ROC) curve analysis.

The ROC curve analysis assessed each miR's diagnostic accuracy and their respective ratios (Figure 3). The area under the ROC curve (AUC) was used as diagnostic index (AUC: miR-let7a = 0.76, miR-34c-5p = 0.53, and miR-21-5p = 0.56; miR-21-5p/miR-let7a = 0.81, miR-34c-5p/miR-let7a = 0.68, and miR-21-5p/miR-34c-5p = 0.62). The miR-21-5p/miR-let7a ratio showed the best AUC value (AUC = 0.81) (Figure 3).

### 3.1. Evaluation of the Prognostic Role of miRs Expression on Recurrence

**Figure 4:**
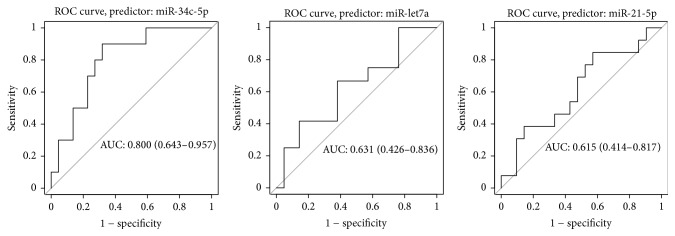
Prognostic accuracy of miRs in laryngeal squamous carcinoma, according to ROC curve analysis.

The prognostic accuracy/significance of the miRs on disease recurrence following surgical resection was assessed with ROC curve analysis (AUC: miR-let7a = 0.631, miR-34c-5p = 0.800, and miR-21-5p = 0.615) (Figure 4). Since miR-34c showed a good prognostic value, the optimal cut-off value was determined according to the best ROC curve sensitivity and specificity (best cut-off miR-34c-5p = 0.0305 relative expression in a.u., sensitivity = 0.90, 1 − specificity = 0.32).

### 3.2. Disease-Free Survival

**Figure 5:**
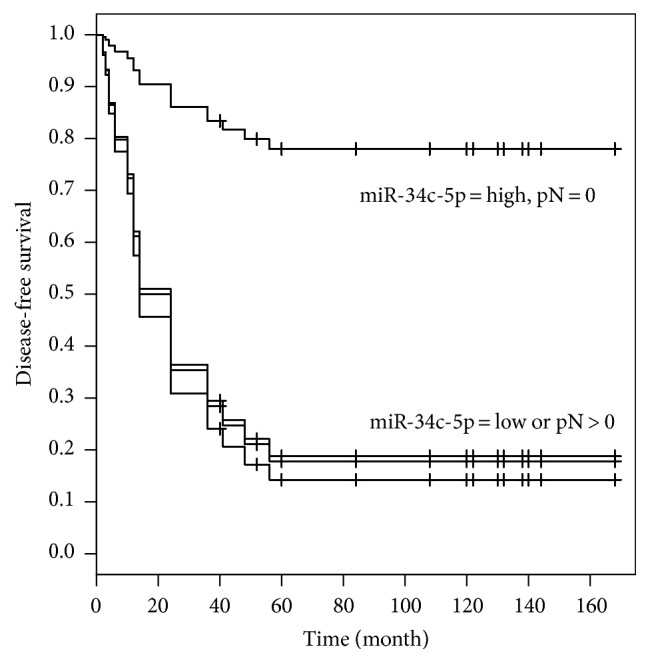
Disease-free survival stratified for miR-34c-5p and pN, according to Kaplan–Meier estimates.

At univariate analysis, miR-34c-5p levels (low level if miR-34c-5p < 0.0305, high level otherwise) (*p* = 0.003), lymph node metastasis pN (pN = 0 versus pN > 0) (*p* = 0.009), and tumor grade of differentiation G (G1 versus G2 or G3) (*p* = 0.02) were identified as significant prognostic predictors for disease-free survival (Figure 5). Multivariate analysis by Cox proportional hazard revealed that only pN and miR-34c-5p with the lowest AIC remained in the multiple model.

Significant prognostic predictors for disease-free survival were miR-34c-5p (hazard ratio = 7.830, 95% confidence interval 2.225 to 27.552) and pN (hazard ratio = 6.926, 95% confidence interval 1.928 to 24.887) (Table 2).

### 3.3. Overall Survival

**Figure 6:**
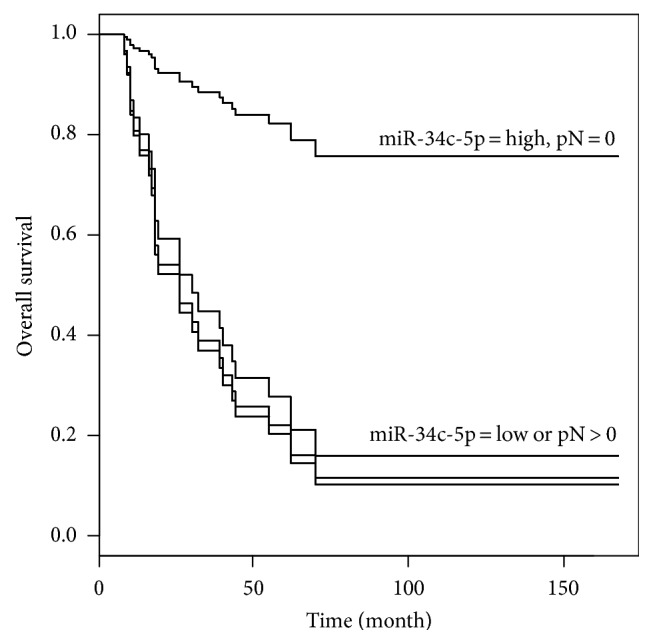
Survival function stratified for miR-34-5p and pN, according to Kaplan–Meier estimates.

At univariate analysis, miR-34c-5p levels (*p* = 0.002), lymph node metastasis pN (*p* = 0.003), radiation therapy, RT (*p* = 0.0004), and chemotherapy (*p* = 0.01) were identified as significant prognostic predictors for overall survival (Figure 6). Multivariate analysis (Table 3) revealed miR-34c-5p (hazard ratio = 7.32, 95% confidence interval 2.33 to 23.00) and pN (hazard ratio = 7.45, 95% confidence interval 2.28 to 24.30) as significant prognostic factors for OS.

## 4. Discussion

There has been increasing evidence that implicates miRNAs in the carcinogenic process [23–27] reporting that over 50% of miRNAs are located in cancer-associated genomic regions or in fragile sites [27]. Significant associations have been reported between miRNA profiles and essential tumor features and patient survival [28–30].

The expression patterns and biological functions of microRNAs have recently been investigated in LSCC, suggesting interesting potential novel therapeutic options [17, 31–34]. Based on previously published results associating disease malignancy and miR-34c-5p expression and p63 and Ki-67 immunostainings in LSCC survival [17, 35–39], we endeavoured to investigate miR-21-5p and let-7a expression in LSCC. The simultaneous analysis of three miRs (miR-34c-5p, miR-21-5p, and let-7a) in the same patient sample allowed us to optimize the evaluation of their prognostic and diagnostic significance, disentangling the potential different roles of the three miRs in the progression of the diseases. This is the first report to comprehensively measure miR-34c-5p, miR-21-5p, and let-7a in patients affected by LSCC.

The downregulation of miR-34c-5p in tumor tissue was related to worsened disease-free and overall survival. This finding suggests that low miR-34c-5p expression is an independent predictor of poor survival for LSCC patients and may be linked with increased tumor aggressiveness. Therefore, the authors hypothesize that miR-34c-5p may be pivotal in LSCC progression, invasion, and metastasis and could be a promising predictive marker for patient relapse. These results are supported by previous published data highlighting that the miR-34 family, including miR-34a and miR-34b/c, acts as a tumor suppressor, able to inhibit different human tumor growth and invasion [36–39], including human LSCC [31–34]. Although previous data have already reported the association of miR-34c-5p to LSCC recurrence [17], the data in the current study present for the first time the association of miR-21-5p and miR-let-7a and the comparison between the clinical relevance of these miRs and that of miR-34c-5p in LSCC development, progression, and recurrence.

The current study also shows that downgraded level expression of miR-let-7a is significantly associated with tumor tissue compared to normal, adjacent tissue. However, the most relevant finding was that the downregulation of let-7a was associated with stage *N*, with the minimum levels of expression in the samples *N* > 0. These data show that let-7a may have a prognostic value in distinguishing LSCC with or without a predisposition towards lymph node metastasis.

Furthermore, the downregulation of let-7a expression found in nondifferentiated tumors compared to both moderately differentiated and well-differentiated tumors indicates its potential role in LSCC cell differentiation. These results are in accordance with previous studies suggesting that miR-let-7a may act as a tumor suppressor (it is poorly expressed in lung and colon cancer) [38, 39] and its downregulation has been reported to have a prognostic impact on the survival of surgically treated lung cancer patients [38].

MiR-21-5p has been one of the most extensively studied miRNAs, and its role in carcinogenesis and several confirmed targets is clear [40, 41]. MiR-21-5p has been heralded to function as an oncogene because it is overexpressed in many types of tumors compared with normal tissues [42, 43], including head and neck cancers [44]. Importantly, as an oncogenic miRNA, miR-21-5p may be responsible for invasion and metastasis along with tumor growth. These effects are probably associated with multiple tumor suppressor genes' concurrent downregulation throughout the various stages of tumor progression, including PDCD4, TPM1, maspin, and PTEN [20, 45].

With reference to miR-21-5p, our data showed its significant overexpression in LSCC tumor tissues compared with the corresponding normal tissues. The findings appear to be in line with the observations of other recent miR-21 dedicated studies [46, 47].

Finally, in order to improve the predictive potential for individual microRNA alterations, we used expression ratios as described by Gordon et al. [22]. Interestingly, the ratio between the miR-21-5p and miR-let-7a expression levels seems to have a greater discriminatory power compared to each miRNA, taken individually to distinguish tumor tissue from normal tissue. A 36-fold increase of miR-21-5p/let-7a ratio was observed in LSCC samples compared to the adjacent healthy tissue. These data suggest that these microRNAs' expression ratios could potentially assist in a simple and early diagnosis for LSCC. Further studies are necessary including independent LSCC tumors and their expression ratios in different stage tumors along with screening samples (such as saliva and mouthwash), to clarify the true clinical applicability of these findings.

The current study has some important limitations. Firstly, the study comprises a small patient cohort, and secondly, this is a retrospective study and the results have to be confirmed by a prospective study. A sensitivity analysis could not be performed, due to sample size (*n* = 43). Another major limitation of this study is related to the involved subsite.

The current patient cohort exclusively included subjects with LSCC. However, considering that the surgical margin and lymph node status might be two of the most fundamental factors affecting outcome, our evaluation included only patients without a tumor in or near (<1 mm) the surgical margins or extracapsular lymph node spread (ECS).

Finally, as aforementioned, the authors' basic understanding of miRNA target recognition was a significant handicap to understanding pathways of cellular signaling and connections between downregulated miRNAs in cancer. Moreover, as shown by some recent reviews [48–51], at the present time there are also discordant data concerning the real prognostic value of some important molecular biomarkers in LSCC and, until now (except for HPV), no substantial clinical impact has been documented. This lack of concordance could be connected to molecular mechanisms that include miRNAs patterns and their functions to inhibit or activate oncogenes and tumor suppressors. Interestingly, previous reports have shown that miRNAs enhance malignant tumor phenotype by inhibiting various tumor suppressor genes' expression simultaneously [52].

Therefore, in addition to the above-mentioned genes, we considered that there might be other target genes of miR-21-5p, miR-let-7a, and miR-34c-5p in LSCC which are still to be discovered and the exact mechanism by which these miRs and their possible target genes are associated with LSCC development warrants further research.

## 5. Conclusions

MiR-21-5p, miR-let-7a, and miR-34c-5p seem to play a pivotal role in LSCC carcinogenesis. The miR-21-5p/let-7a ratio may hold a significant clinical diagnostic potential to distinguish tumor tissue from normal tissue, while miR-let-7a levels may assist in predicting lymph node metastases and miR-34c-5p could prove to be a critical biomarker for patient outcome. Once validated by prospective clinical trial results, these observations may have important implications for LSCC individualized patient treatment and care.

## Figures and Tables

**Table 1:** Overview of the clinical and pathological characteristics of patients with primary LSCC (*N* = 43).

	Years (SD)
Mean age	66.51 (8.02)

	*Number of patients (%)*

Sex	
Male	42 (97.67)
Female	1 (2.33)

Subsite	
Supraglottic	8 (18.60)
Transglottic	33 (76.74)
Subglottic	2 (4.65)

Type of surgery	
Total laryngectomy	33 (76.64)
Partial laryngectomy	10 (23.26)

RT after surgery	
Yes	22 (51.16)
No	21 (48.84)

CHT after surgery	
Yes	10 (23.26)
No	33 (76.74)

Grading	
G1	5 (11.63)
G2	29 (67.44)
G3	9 (20.93)

pT	
T3	31 (72.09)
T4	12 (27.91)

pN	
N0	27 (62.79)
N1	3 (6.98)
N2	13 (30.23)
N3	0

pM	
M0	43 (100)
M1	0 (0)

Relapse	
Yes	23 (53.49)
No	20 (46.51)

**Table 2:** Coefficients of the variables included in the Cox proportional hazard model for disease-free survival with lowest AIC.

Variable	Coeff.	SE	*Z*	*p*
miR-34c-5p	2.058	0.642	3.206	0.001
pN	1.935	0.653	2.966	0.003
miR-34c-5p:pN	−2.089	0.864	−2.417	0.016

**Table 3:** Coefficients of the variables included in the Cox proportional hazard model for overall survival with lowest AIC.

Variable	Coeff.	SE	*Z*	*p*
miR-34c-5p	1.99	0.584	3.41	<0.001
pN	2.01	0.603	3.33	<0.001
miR-34c-5p:pN	−2.18	0.801	−2.72	0.007
